# MetNetAPI: A flexible method to access and manipulate biological network data from MetNet

**DOI:** 10.1186/1756-0500-3-312

**Published:** 2010-11-18

**Authors:** Yves Sucaet, Eve Syrkin Wurtele

**Affiliations:** 1 Department of Genetics, Development and Cell Biology, Iowa State University, Ames, IA 50011, USA; 2 Interdepartmental Program in Bioinformatics & Computational Biology, Iowa State University, Ames, IA 50011, USA

## Abstract

**Background:**

Convenient programmatic access to different biological databases allows automated integration of scientific knowledge. Many databases support a function to download files or data snapshots, or a webservice that offers "live" data. However, the functionality that a database offers cannot be represented in a static data download file, and webservices may consume considerable computational resources from the host server.

**Results:**

MetNetAPI is a versatile Application Programming Interface (API) to the MetNetDB database. It abstracts, captures and retains operations away from a biological network repository and website. A range of database functions, previously only available online, can be immediately (and independently from the website) applied to a dataset of interest. Data is available in four layers: molecular entities, localized entities (linked to a specific organelle), interactions, and pathways. Navigation between these layers is intuitive (e.g. one can request the molecular entities in a pathway, as well as request in what pathways a specific entity participates). Data retrieval can be customized: Network objects allow the construction of new and integration of existing pathways and interactions, which can be uploaded back to our server. In contrast to webservices, the computational demand on the host server is limited to processing data-related queries only.

**Conclusions:**

An API provides several advantages to a systems biology software platform. MetNetAPI illustrates an interface with a central repository of data that represents the complex interrelationships of a metabolic and regulatory network. As an alternative to data-dumps and webservices, it allows access to a current and "live" database and exposes analytical functions to application developers. Yet it only requires limited resources on the server-side (thin server/fat client setup). The API is available for Java, Microsoft.NET and R programming environments and offers flexible query and broad data- retrieval methods. Data retrieval can be customized to client needs and the API offers a framework to construct and manipulate user-defined networks. The design principles can be used as a template to build programmable interfaces for other biological databases. The API software and tutorials are available at http://www.metnetonline.org/api.

## Background

Analysis of the topology of biological networks provides understanding of structure, function and interaction among cellular entities [1]. As knowledge and understanding of living systems expands, biological network databases are becoming increasingly sophisticated, in terms of data complexity and overall functionality. To facilitate integration with various bioinformatics software packages, many online pathway and network databases offer static data download and conversion methods [2]. Several larger databases, including KEGG [3], BioCyc [4] and Reactome [5], also offer Application Programming Interfaces (APIs). These resources are used by the community to incrementally enrich datasets, such that each iteration is better and more complete than the previous one.

The MetNet systems biology platform is a suite of software programs that model metabolic and regulatory pathways in plants [6]. At its core is MetNetDB, which represents an integrated pathway-database for plant species and combines various data sources such as AraCyc [7], TAIR [8], AGRIS [9], and atPID [10]. The database allows users to integrate pathways and interactions and keep track of entities of interest in a customizable way.

Through a public website http://www.metnetonline.org, users control various network resources including 1) the ability to bookmark pathways, 2) tracking user-defined components of interest, and 3) a localization datalayer. Users can create new pathways by combining and modifying existing pathways and then save the new pathways. Using the website, pathways can be exported to SBML (for visualization with CellDesigner [11]) or XGMML (for visualization with Cytoscape [12]). All the above functions are now available through our API. MetNetAPI is an alternative interface to the MetNet plaform for tasks that are not easily performed with already existing software tools, time-consuming or repetitive.

An API allows data to be approached and viewed in several modi. Unlike statically-exported files such as data dumps and standardized schema which offer only a single view of the data, whereas an API enables much more user customization, such that a researcher can view or computationally manipulate the data in multiple ways. Consider the static SBML BioModels dataset, wherein each file represents a single pathway [13]. Assume someone downloads this dataset and wants to gain a more complete understanding of it by creating a list of all the molecular entities that participate in all the pathways. This list can then in turn be used to connect pathways with overlapping components (e.g. pathways in which starch participates can be combined to study starch metabolism and its regulation [14]). However, composing a complete list of entities that make up all the pathways in which starch participates entails writing a piece of custom parsing software. In contrast, an API can implement a method that automatically extracts a list of participating entities for a collection of the pathways in which they occur.

## Implementation

### The choice of an API

Several options exist to share information contained in a biological database. One option for transfer of database content is a data dump. This exposes all the information contained in the database. However, it may require significant effort to understand (and possibly reconstruct) the original database schema.

A second option is to support a standard data format. Chado and BioSQL are two examples of standardized data schema specific to sequence databases [15]; BioPax, SBML and PSI are the most widespread file formats for representation of biological networks [16]. Each standard has its own set of limitations as to what types and resolution of data it can represent. Supporting multiple formats is time-consuming.

A third option is providing an Application Programming Interface (API). A major advantage is that content and functionality are combined [17]. Tying an API directly to a biological database has been done by other groups. MetaCyc [18] is based on the Lisp programming language and interfaces with local MetaCyc-derived databases, while MetNetAPI offers broader programming language support and always connects to the remote "live" MetNetDB database. BioMart [19] is a generic biological repository, and configuring it to support complex network data takes a long time. Its general-purpose nature also makes it slow to run complex queries due to the meta-data that needs to be interpreted first. KEGG [20] and Reactome [21] offer a webservice interface, based on XML and SOAP/REST. A webservice can be considered as a special type of API and provides its own particular problems: A wrapper must be provided around the webservice to facilitate communication and data exchange. This effectively means a secondary API has to be provided to communicate with the initial API. Even as this process can often be automated (through frameworks such as JAX-WS http://jax-ws.dev.java.net/, Axis http://ws.apache.org/axis/ or XFire http://xfire.codehaus.org/), it is far from efficient. REST-based webservices are somewhat less cumbersome in this regard (they are lightweight, produce human readable results, and require no toolkits like SOAP does), but they have their own peculiarities: Every resource needs to be accessible through a unique URI. This means that information is represented in a hierarchy, which can become complicated very quickly and cumbersome to browse. It is possible, however, to circumvent this problem by allowing querying of the dataset at a different location on the website. The URL to a REST-resource is then a query-string in its own right. While the messaging protocol involves less overhead than its SOAP-counterpart, the lack of required message meta-data makes these environments at the same time less intuitive and harder to query for complex data. Reactome is one such pathway database [21] that supports REST through BioMart's MartService [19]. Doodle is another resource that supports REST [22], while GenMAPP [23], WikiPathways [24] and CPDB [25] choose to provide SOAP-based services.

If functionality is added to the webservice (either REST or SOAP), supplemental resources - CPU, memory, hard disk - for the server hosting the service must be considered. Webservices therefore seem to be destined to either offer limited functionality (and thus be less useful), or offer extensive functionality but artificially limit access to them because no institution can gather unlimited bandwidth and resources to serve the world. MetNetAPI offers close proximity (strong datatyping) to the MetNetDB database and underlying model, while still able to provide flexibility and abstraction in regard to biological information content. Processing of information mostly occurs on the client running the API, which results in a more distributed load. This presents opportunities to better plan (and distribute) resources across various projects.

### API implementation

MetNetAPI is designed as an object model that abstracts and encapsulates the data in the underlying MetNetDB repository. We chose Java, R and Microsoft.NET as target programming languages because they are platform independent and are widely in use today. Users do not need to understand the internal intricacies of the back-end database model. The goal is to hide complex data modelling techniques and allow the bioinformatics software developer (and by extension the biologist) to get started using novel integrated datasets quickly. Overhead is kept to a minimum, as there is no WSDL-file to be parsed, as with SOAP. The structure of the information in MetNetAPI is exposed intuitively through Java reflection mechanisms that are provided in most development environments.

MetNetAPI is a Java jar-file (or .NET Assembly) which contains several logically-ordered namespaces (abstract containers that express semantic categories of code). The main namespace is edu.iastate.metnet (Edu.Iastate.Metnet in .NET). Underlying namespaces and classes allow reasoning by type and allow a programmer to bring biological semantics into the program code. This is in contrast with many other APIs, which result in generic Dictionary-objects, which still require further interpretation and parsing after retrieval. The same argument applies to webservices (especially REST), where the returned output is text-based that requires further processing.

Querying of MetNetDB through MetNetAPI is optimized for efficient memory use. Similar to the Lazy Load concept in the Java persistence library Hibernate http://www.hibernate.org, we adapted Just In Time (JIT) compilation for data retrieval. When retrieving a pathway, only the main data is obtained from the database. Information represented in linked tables (one-to-many or many-to-many) is retrieved when the respective methods are invoked. This occurs transparently, so a client application should function optimally and use a minimal footprint whether retrieving a list of "all pathways", or constructing an integrated network from "all amino-acid biosynthesis-related reactions". The JIT data retrieval mechanism not only encapsulates a complex data model, it also makes retrieval and reconstruction of network data efficient. This behaviour is impossible to implement through webservices, as the server cannot "guess" what clients want to do with a returned piece of information in the future. One option would be to provide a verbose-like parameter when calling the webservice, which introduces additional overhead for the programmer consuming the service. Another option would be that the server assumes a worst-case scenario and streams all available (hierarchical) information back to the client, leading to increased (and possibly unnecessary) server-load and network traffic.

## Results

### MetNetAPI

MetNetAPI is a flexible API that interacts with and retrieves data from MetNet, an established information resource and suite of software applications for model organisms, currently including Arabidopsis, soybean and grapevine http://www.metnetonline.org[6]. By accessing MetNet infrastructure, the researcher can obtain integrated metabolic and regulatory biological network data, in addition to other new layers of information that were not previously available in any central location.

The API allows a software developer to navigate the database from multiple points of view, without having to understand the underlying database schema. The database can be navigated either as a list of pathways, a list of entities or a collection of organism-centric networks. In contrast, static data files allow only one such point of view and require customized parsing to determine the answers to specialized questions. Examples of user queries would include "which elements in a list of entities participate in at least two pathways" or "for a given collection of pathways, single out and reconstruct a regulatory network". MetNetAPI can answer such queries without extensive programming for any respective list of entities (e.g., genes, RNAs, polypeptides, protein complexes, metabolites, or combinations thereof). The API approach allows a database platform to abstract and expose its repository data, along with its functionalities.

### Core classes

The MetNetAPI is designed to capture MetNet architecture, which centers around four central classes:

An *Entity *represents any type of molecular entity that can be found in a biological environment. Entities have a general categorical descriptor that describes the type of an entity, such as "gene", "RNA" or "Protein Complex". They can be organism-specific (in the case of a gene) or not (universal metabolites such as ATP or glucose).

A *LocalEntity *represents a particular entity found within a sub cellular location. An example is the molecule (Entity) ATP, which is found in several compartments (locations) in the cell, including mitochondrion, nucleus, plastid, and cytosol. Therefore, the Entity ATP has four associated LocalEntities.

An *Interaction *represents the impacts or transformations among entities. Due to the diversity and generalization of the Entity class, Interactions are kept equally generic. Like entities, they are classified. Interactions include enzymatic reactions, transport, transcription, translation, and various classes of regulatory inhibition and activation such as allosteric effector or indirect positive regulation.

A *Pathway *represents a group of multiple interactions and the associated biomolecules organized into a convenient functional unit. The pathway concept in MetNetAPI is defined as an unordered collection of Interaction objects. In order to allow developers to determine the start- and end-points of a pathway, getSources() and getSinks() methods are provided.

Peripheral classes are provided to further define pathways and represent MetNet-specific data. The *Organism *class represents information about organisms currently in MetNetDB. *EntityType *and *InteractionType *represent the different types of respective entities and interactions. *PathwayClass *provides a Pathway Ontology to navigate through the collection of all pathways, which is based on AraCyc pathway classes. *CellLocation *provides a similar hierarchy that can be used as an alternate pathway ordering tree.

Pathways are arbitrary groupings of interactions. Even for well-defined pathways such as glycolysis and TCA cycle, different views can be created, which may or may not include the genes and the transcriptional and regulatory framework of the various enzymes involved. As more knowledge is acquired through scientific experimentation, pathways may become so complex that it is beneficial to break them into smaller units for some applications. Conversely, smaller pathways may be joined into a larger unit or a super-pathway for meta-analysis.

To model these evolving datasets, a *Network *class is provided. It serves the purpose of providing custom granularity. A *Network *object consists of a custom collection of interactions. A *Network *incorporates the concept of a pathway, yet it is not confined to the boundaries of a predefined pathway. Networks can be constructed either by combining existing pathways or by adding individual interactions.

**Figure 1:**
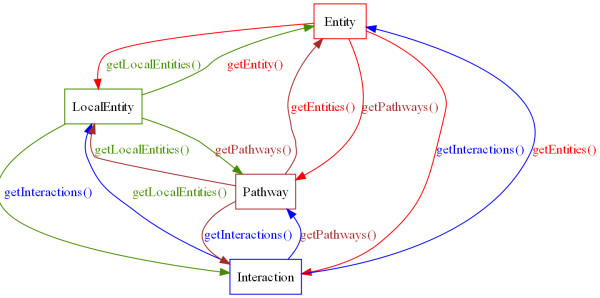
**Interconnectivity between the API's classes**. All core classes in MetNetAPI are interconnected. This allows for upward and downward navigation (e.g. one can as easily ask "what entities make up a particular pathway", as "what pathways does a particular entity participate in").

Several APIs offer top-down approaches to network data. An example is libSBML, in which a pathway consists of reactions, which consist of molecular species [26]. It is currently not possible through libSBML to work backward (e.g. to see which interaction a molecular species participates in). MetNetAPI offers easy navigation and conversion between all its core classes (see Figure 1). This makes it particularly easy to write p-neighbourhood applications, where one is interested in examining the connectedness between network components.

### Searching and filtering

Most all network database websites have a search-function. Upon downloading files for offline use, the online functionality is no longer available. This means that a data dump does not always offer the correct amount of information one is interested in. Much effort needs to be invested in study of the original data format and writing parser code to extract the information of interest.

Through MetNetAPI, online search-capabilities are extended and can be integrated in desktop and other applications (these do still need to have network-connectivity to allow communication between the API and our back-end database). This makes it convenient to execute a large number of queries against MetNet. The investigator can automatically determine which pathways a given list of metabolites participates in, restrict a pathway to its regulatory interactions, or request a list of affected pathways for a set of up-regulated genes. Most Java-classes in the MetNetAPI library have a static search() method, which allows developers to launch queries against MetNetDB in real time, without having to go to a website, fill out a form and submit it.

Filtering using MetNetAPI is similar to searching, but zeros in on results within results. For example, a user could extract all gene regulatory interactions from a previously defined set of pathways (combined as a Network object). Alternatively, a user could look at a complex pathway with 100+ interactions, and decide to remove temporary clutter caused by transcriptional and translational events. The resulting "core" pathway makes it easier to understand the metabolic functions performed by the pathway.

### Applications

The availability of a dynamic code-driven class hierarchy instead of a collection of static, rigid files allows developers to rapidly provide MetNet data and bring its functionality to their own applications. MetNetAPI is object-oriented, which allows for code to be mixed with data (methods and properties). When a collection of pathways is represented by a PathwayVector object, functions to manipulate the member objects are provided. This is preferable to the use of rigid files, or the passing back and forth of Dictionary-like structures.

**Figure 2:**
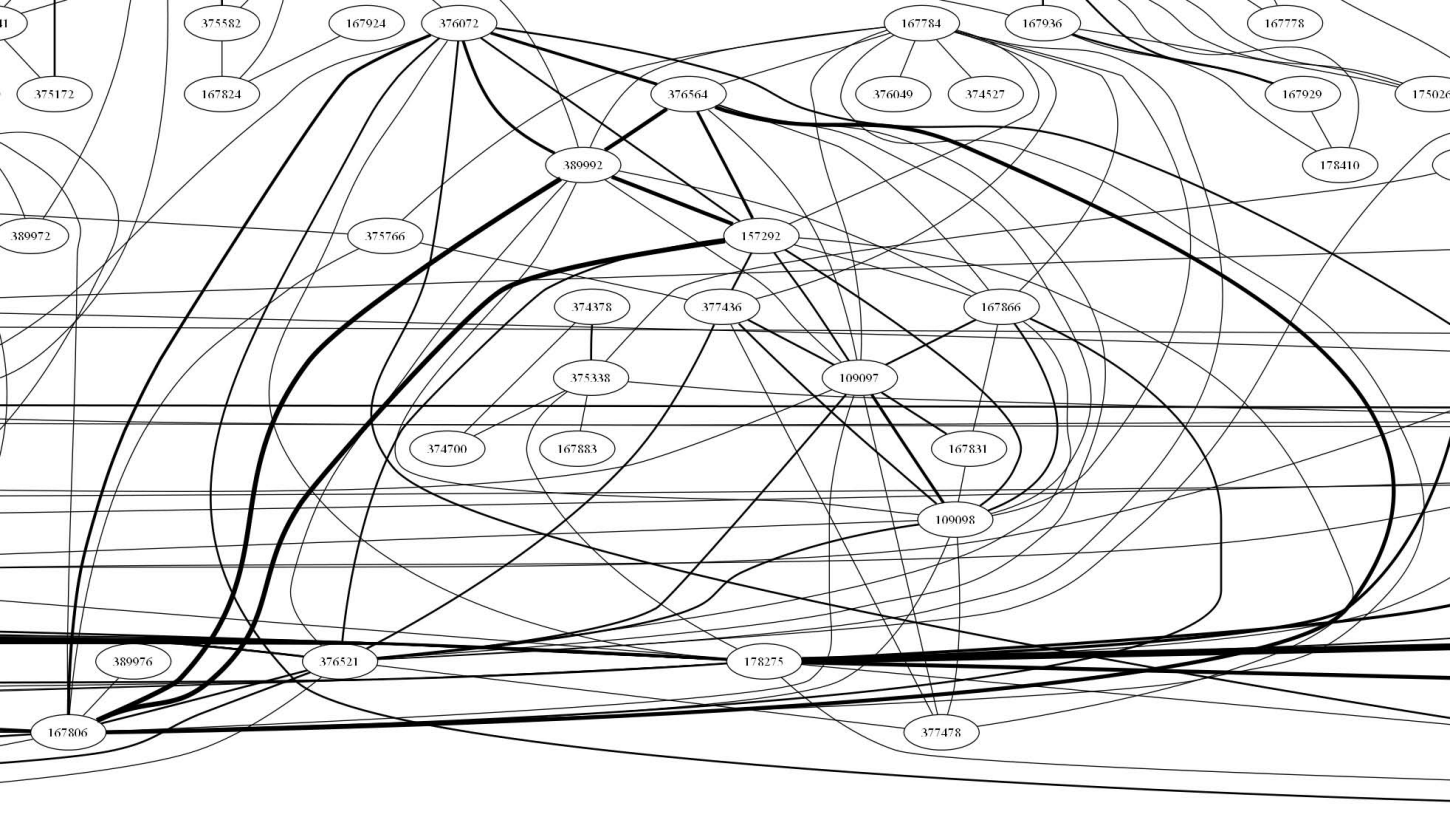
**Details of a map that illustrates shared genes between pathway**. With MetNetAPI, it is straightforward to compute a distance matrix between a set of pathways. The matrix can then be visualized with a tool like GraphViz (thicker lines indicate a closer distance). Details of the visualized matrix are shown here; the full rendering is available in Supplemental file distance.png.

Source code is provided [Additional file 1] that creates a distance matrix among all 403 pathways in the database. The algorithm results in a GraphViz-compatible http://www.graphviz.org .dot-file, details of which are shown in Figure 2. The complete rendered .png-file is available as [Additional file 2]. Additional examples are available on the MetNetAPI tutorial website.

MetNetAPI exports data to standard data formats such as SBML or XGMML (used in Cytoscape). This functionality is available for developers that wish to exploit the richness of MetNetDB. It also allows integration of MetNet-originated data into a more expansive research pipeline. The Network class contains a set of methods that allow export to a variety of standards. To ensure compatibility with a wide spectrum of software, the depth of information has been restricted to a minimum. So, while the Network class is recommended to prepare data for external software such as Jarnac (SBML) or Cytoscape (XGMML), specialized needs would require a developer to generate customized export-routines.

MetNetAPI facilitates the creation of static files based on dynamic actions. An example would be to gather the 5 pathways in the database that describe the metabolism and signalling associated with the plant hormones brassinosteroids and auxins into a single Network object, and to export this network to a single XGMML file. This file can be directly imported into Cytoscape to enable visualization and further analysis of a user-specified unit of biology (eliminating the need to import multiple files that represent individual pathways).

### Initial adaptations

**Figure 3:**
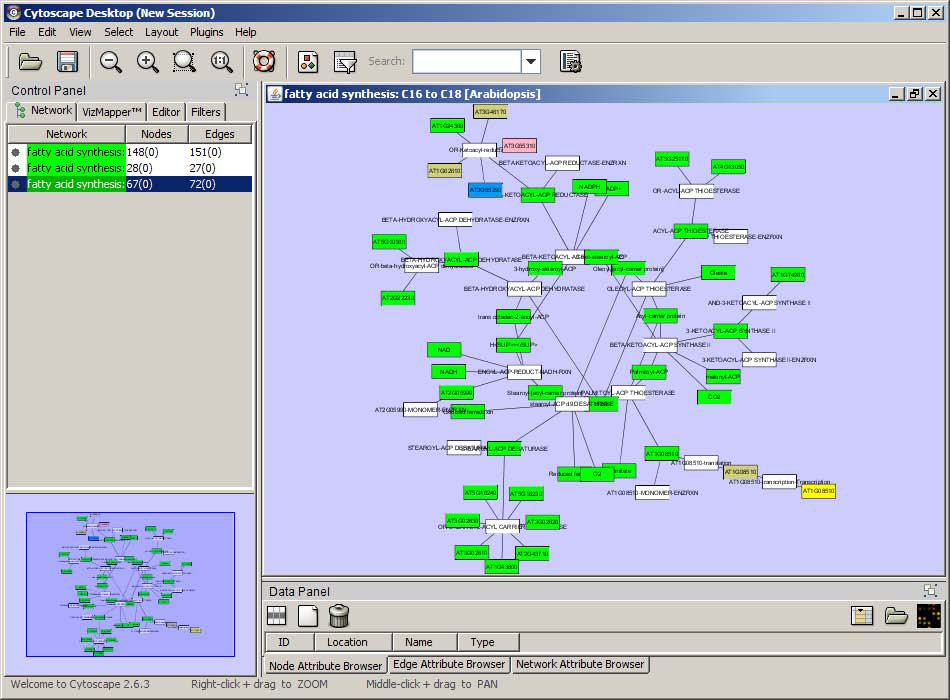
**Cytoscape plugin developed with MetNetAPI**. As a proof of concept, a Cytoscape plugin was developed that brings pathway data along with localization information into the Cytoscape environment.

Several proof-of-concept applications using MetNetAPI have already been developed: We have developed the MetNetScape plugin to allow a user to select an organism and pathway to be imported into Cytoscape. An example of an imported pathway is shown in Figure 3. The plugin is available through our website http://www.metnetonline.org/api/cytoscape/ and source code is available upon request so its functionality may be extended.

A more complex plugin has been developed for CellDesigner [11] to allow exchange and integration of BioCyc and MetNet pathways. The plugin uses the edu.iastate.metnet.edit namespace to publish new pathways in MetNetDB. This makes MetNet useful as a community annotation platform. The plugin allows for seamless one-click publication of newly constructed pathways into MetNet [27]. It is being used to bring manually-constructed grapevine pathways [28] into MetNetDB.

MetaOmGraph (MOG) is an application to display large expression datasets [6,29]. Subsets of entities (genes or metabolites) can be selected in MetNet based on user-specified criteria. These lists can be sent to MOG for further analysis via a user's MetNet profile (a free personal account created through our website [30]). Integration works both ways: genes can be selected in MOG and published to a personal MetNet account [31].

Large biological networks often benefit from visualization in 3D [32]. Walrus http://www.caida.org/tools/visualization/walrus/ is a desktop-application to visualize 3D-data. A proof-of-concept application has been developed that enables a user visualize MetNet pathways in 3D on a standard computer [33]. The application retrieves data through MetNetAPI to compute the optimal spanning tree to be used by Walrus to create the environment.

MetNetGE [34] is an environment that uses Google Earth infrastructure to produce layered representations of pathways in MetNet. Pathways are visualized as stacked planes, whereby each plane represents a certain type of entity (genes, RNA, polypeptides, or metabolites). MetNetGE uses MetNetAPI to retrieve pathway ontology data and gene information.

## Discussion

We have adopted the API as a method to standardize development of applications that exploit the MetNetDB dataset. In addition to facilitating prototyping and rapid application development, this approach ensures consistency across end-user interfaces, command line interfaces, and graphical user interfaces. MetNetAPI is flexible and can be modified, based on needs of internal and external software developers.

We are exploring the possibilities of using the API in environments other than Java. This has already lead to integration of MetNetAPI into Microsoft .NET and R http://www.r-project.org through the rJava bridging software http://www.rforge.net/rJava/.

Advanced programming knowledge (such as SQL or JDBC) is not required for using MetNetAPI. The complexity of the underlying data model is encapsulated within the API. The interface is only slightly less universal than the socket-based protocol provided by BioCyc [18], and the choice of Java allows the API to be used by a broad audience of software developers and bioinformatics researchers. Importantly, unlike a socket-based approach, installation and troubleshooting of MetNetAPI is easy, since it relies on basic Java coding practices. MetNetDB represents a large complex metabolic and regulatory network and contains multiple interaction types, kinetic information, and manually curated subcellular localization assignments.

## Conclusions

Online databases often provide data export by means of static downloadable files or dynamic webservices. MetNetAPI provides an additional approach to data export. The API provides a method to standardize development of applications that exploit MetNetDB, but may also serve as a framework and template for other pathway databases. A standardization of terminology among different databases would certainly benefit developers that work on integrative applications. Many databases expose similar types of data, and the definition of a minimal set of interfaces that pathway database APIs may be expected to implement would be helpful. MetNetAPI can be a first step in this direction.

Apart from facilitating prototyping and rapid application development, our approach ensures consistency and data integrity across command line interfaces and graphical user interfaces alike. The choice of Java and Microsoft.NET allows the API to be used by a broad audience of software developers and bioinformaticists. The complexity of the underlying data model is encapsulated within the API. Because it is a Java-API rather than a webservice, more functionality can be provided without requiring extensive computational resources on the server-side.

For a densely populated and information-rich database (such as MetNetDB), our API model offers many advantages. It has the ability to incorporate online search capabilities into custom-built applications. It also offers the option to customize the granularity of pathways of interest. MetNetAPI captures user-defined network structures into self-contained semantic objects. Through *Network *objects, combinations of existing or putative novel pathways can easily be constructed, manipulated and refined. MetNet is an information resource, as well as an active toolkit to develop new hypotheses. Many complicated operations, which would be difficult to implement via xml or text-based files, can be accomplished through MetNetAPI. These feature flexible capabilities to agglomerate data over multiple pathways, to examine connectivity among different datatypes, and prepare custom datasets for use in other downstream applications. MetNetAPI is fully documented, free of charge and can be downloaded from http://www.metnetonline.org/api/cytoscape/.

## Availability and requirements

Project name: MetNetAPI

Project home page: http://www.metnetonline.org/api

Operating system(s): Platform independent

Programming language: Java, Microsoft.NET, R

Other requirements:

Java-specific: Java 1.3.1 or higher, MySQL JDBC driver

Microsoft.NET-specific: Microsoft.NET 1.1 or higher, MySQL .NET driver

License: None

Any restrictions to use by non-academics: No

## Abbreviations

API: Application Programming Interface; CSV file: comma-separated values file; KEGG: Kyoto Encyclopaedia of Genes and Genomes; MOG: MetaOmGraph; REST: REpresentational State Transfer; SBML: Systems Biology Markup Language; SQL: Structured Query Language; SOAP: Simple Object Access Protocol; URI: Uniform Resource Identifier; WSDL: WebService Description Language; XGMML: eXtensible Graph Markup and Modeling Language.

## Competing interests

The authors declare that they have no competing interests.

## Authors' contributions

YS carried out API design and development. ESW conceived of the study, and participated in its design and coordination and helped to draft the manuscript. All authors read and approved the final manuscript.

## Supplementary Material

Additional file 1 **Sample program code to generate an inter-pathway map**. Sample program that creates a distance matrix between a set of pathways. The distance is based on the number of genes that are shared between two pathways. Output is sent to a .csv-file as well as a GraphViz-compatible .dot-file. A rendering of the .dot-file is provided as a separate Supplemental file. Click here for file

Additional file 2 **Inter-pathway map based on shared genes between pathways**. A complete rendering of the distance matrix that was computed with the sample program provided in distance.java. Click here for file
